# Genome Sequence of Torulaspora microellipsoides CLIB 830^T^

**DOI:** 10.1128/genomeA.00615-18

**Published:** 2018-06-28

**Authors:** Virginie Galeote, Frédéric Bigey, Hugo Devillers, Raúl A. Ortiz-Merino, Sylvie Dequin, Kenneth H. Wolfe, Cécile Neuvéglise

**Affiliations:** a SPO, INRA, SupAgro, Université Montpellier, Montpellier, France; b Micalis Institute, INRA, AgroParisTech, Université Paris-Saclay, Jouy-en-Josas, France; c UCD Conway Institute and School of Medicine, University College Dublin, Dublin, Ireland

## Abstract

We report here the genome sequence of the ascomycetous yeast Torulaspora microellipsoides CLIB 830^T^. A reference genome for this species, which has been found as a donor of genetic material in wine strains of Saccharomyces cerevisiae, will undoubtedly give clues to our understanding of horizontal transfer mechanisms between species in the wine environment.

## GENOME ANNOUNCEMENT

The genus Torulaspora belongs to the Saccharomycotina subphylum and is now composed of eight species (T. delbrueckii, T. globosa, T. franciscae, T. pretoriensis, T. microellipsoides, T. maleeae, T. quercuum, and T. incana); T. maleeae, T. quercuum, and T. incana have been described in the past decade (1–4). Torulaspora delbrueckii is probably the most widely distributed species, found in nature and in anthropic environments requiring fermentation, such as wine or bread, whereas other species, such as T. microellipsoides, appear more sporadically. However, their presence in fermentation vats may play an important role. Indeed, T. microellipsoides has been shown to be the donor of a DNA region of at least 158 kb to Saccharomyces cerevisiae wine strains, which confers to them an adaptive advantage during wine fermentation (5, 6). Yet, only the T. delbrueckii genome sequence is available (7). Sequencing the genome of T. microellipsoides strain CLIB 830^T^ (=CBS 427) may therefore contribute to an increase in the genomics knowledge of this clade.

Total genomic DNA was used to construct a paired-end (PE) 500-bp insert library and a mate pair (MP) 6-kb insert library. Both libraries were sequenced using the Illumina HiSeq 2000 platform, resulting in raw sequencing coverage depths of 197× (PE) and 177× (MP). Sequencing reads were cleaned using Trimmomatic version 0.32 (8), resulting in a sequencing depth of 319×. A preliminary assembly of all reads was obtained using SOAP*denovo*2 version 2.04 (9). MP reads were mapped with BWA version 0.6.2 (10), and inward read pairs were removed using BamTools version 2.2.3 (11). A second assembly of PE and cleaned MP reads was performed using SOAP*denovo*2, with a k-mer of 61. Gap closing was performed using GapCloser version 1.12 (9). The final assembly was made of 46 scaffolds (*N*_50_, 1.2 Mb) larger than 1 kb; 8 of them corresponded to mitochondrial DNA. The remaining 38 scaffolds were suitable for automatic annotation.

The structural annotation of protein-coding genes was performed using the Amadea Annotation transfer tool (Isoft, France), with the Lachancea kluyveri CBS3082^T^ genome as a reference (revised version available at http://gryc.inra.fr [12]). Missing genes were investigated through a BLASTX search against the NCBI RefSeq database, with a comparison to an annotation from the YGAP pipeline (13), and manual curation. In total, 5,239 protein-coding genes were predicted, including 145 pseudogenes. tRNA genes were identified using tRNAscan-SE version 1.3.1 (14). Transposable elements were identified by BLAST with known yeast elements from different families, such as *Ty1-copia*, *Ty3-gypsy*, and *hAT*. A family of 38 *Rover* elements was identified, including intact and degenerate copies and miniature inverted-repeat transposable elements (MITEs) (15). Interestingly, 3 tandem *Rover* elements and the surrounding region encoding 4 proteins in scaffold TOMIS04 were found to be almost identical (99.8% identity over 15,389 nucleotides [nt]) to contig 1098 of S. cerevisiae strain AWRI1631 (16). This clearly indicates that this sequence has been acquired by horizontal transfer into AWRI1631 from T. microellipsoides and not from Lachancea species, as previously thought (15).

A reference genome for this species will undoubtedly give clues to aid in our understanding of the transfer of genetic material between species in the wine environment and the mechanisms involved.

### Accession number(s).

The sequence of the T. microellipsoides genome has been deposited at the European Nucleotide Archive (ENA) under BioProject no. PRJEB7632. The accession numbers of the 46 scaffolds are FYBL01000001 to FYBL01000046. The version described in this paper is the first version.
